# The Implications and Future Perspectives of Nanomedicine for Cancer Stem Cell Targeted Therapies

**DOI:** 10.3389/fmolb.2017.00052

**Published:** 2017-07-21

**Authors:** Vimal K. Singh, Abhishek Saini, Ramesh Chandra

**Affiliations:** ^1^Stem Cell Research Laboratory, Department of Biotechnology, Delhi Technological University New Delhi, India; ^2^Department of Chemistry, University of Delhi New Delhi, India

**Keywords:** CSCs, nanomedicine, immunotherapy of cancer, nanocarrier, drug resistance of CSC, autophagy, tumor suppressor protein p53

## Abstract

Cancer stem cells (CSCs) are believed to exhibit distinctive self-renewal, proliferation, and differentiation capabilities, and thus play a significant role in various aspects of cancer. CSCs have significant impacts on the progression of tumors, drug resistance, recurrence and metastasis in different types of malignancies. Due to their primary role, most researchers have focused on developing anti-CSC therapeutic strategies, and tremendous efforts have been put to explore methods for selective eradication of these therapeutically resistant CSCs. In recent years, many reports have shown the use of CSCs-specific approaches such as ATP-binding cassette (ABC) transporters, blockade of self-renewal and survival of CSCs, CSCs surface markers targeted drugs delivery and eradication of the tumor microenvironment. Also, various therapeutic agents such as small molecule drugs, nucleic acids, and antibodies are said to destroy CSCs selectively. Targeted drug delivery holds the key to the success of most of the anti-CSCs based drugs/therapies. The convention CSCs-specific therapeutic agents, suffer from various problems. For instance, limited water solubility, small circulation time and inconsistent stability of conventional therapeutic agents have significantly limited their efficacy. Recent advancement in the drug delivery technology has demonstrated that specially designed nanocarrier-based drug delivery approaches (nanomedicine) can be useful in delivering sufficient amount of drug molecules even in the most interiors of CSCs niches and thus can overcome the limitations associated with the conventional free drug delivery methods. The nanomedicine has also been promising in designing effective therapeutic regime against pump-mediated drug resistance (ATP-driven) and reduces detrimental effects on normal stem cells. Here we focus on the biological processes regulating CSCs' drug resistance and various strategies developed so far to deal with them. We also review the various nanomedicine approaches developed so far to overcome these CSCs related issues and their future perspectives.

## Introduction

Initial studies to define the characteristics of tumors revealed the presence of a rare population which not only have the self-renewing capacity and proliferate for long periods but could also transfer tumors on transplantation in experimental recipient models (Nowell, 1976; Quintana et al., 2010; Meacham and Morrison, 2013). These cells are termed as cancer stem cells (CSCs) or tumor supporting cells or tumor-propagating cells. Showing further experimentally supported the hypothesis through the transfer of tumorigenic properties of CD34+/CD38− human acute myeloid leukemia cells into severe combined immunodeficiency mice (SCID) (Bonnet and Dick, 1997). Since then CSCs are reported in many types of solid tumor (carcinoma) including brain (Singh et al., 2004), lung (Eramo et al., 2008), breast (Al-Hajj et al., 2003; Pece et al., 2010), colon (Diehn and Clarke, 2006; O'Brien et al., 2007; Ricci-Vitiani et al., 2007), liver (Yang et al., 2008), pancreatic cancers (Li et al., 2007) through similar xenotransplantation experiments in mice. Use of genetically engineered mice model to demonstrate stem cell activities by independent research groups (Skin, intestine, and brain tumors) has provided direct evidence in their support (Chen et al., 2012; Driessens et al., 2012; Schepers et al., 2012). Moreover, the ambiguities raised by some critiques about the presence of stem cell activities in tumor cell through direct evidence in natural settings is well resolved (Reya et al., 2001; Maenhaut et al., 2010; Chen et al., 2012; Schepers et al., 2012). Various studies provide substantial evidence of drug resistance, showing the crucial role of CSCs in both tumor progression and disease relapse (Dean et al., 2005; Eramo et al., 2006; Yu et al., 2007; Dylla et al., 2008; Diehn et al., 2009; Liu Y. P. et al., 2013). The evident central role of CSCs in tumor and their related treatments modalities compelled people to define characteristics for the identification which led to accumulating reports on CSCs phenotypic markers. For example high expression level of drug efflux transporter genes, enhanced activation of anti-apoptotic activities, significantly up-modulated DNA repair activities, slow rate of proliferation (often quiescent) capabilities to program the metabolic processes (Vinogradov and Wei, 2012). It is important to be noticed that current treatment for cancer treatments including chemotherapy/radiotherapy and tumor targeting agents may further enhance the CSCs population and can make it harder to be cured due to increasing spread and survival of them (Eramo et al., 2006; Ma et al., 2008). Thus, there are significant chances of tumor relapse after most tumor treatments with anticancer agents that can kill a bulk amount of tumor cells while drug-resistant CSCs remain unaffected and cause regression (Yu et al., 2012; Flemming, 2015; Li S. Y. et al., 2015). These reports indicate an extreme demand for the more clinical and preclinical studies to define the various characteristics of CSCs and how does CSCs respond to different therapeutic regimes. Present strategies to manage CSCs related cancer relapse imply different approaches such as targeting CSCs specific surface markers, ignition of ATP-binding cassette (ABC) transporters blocking, downregulating CSCs self-renewal and survival signaling pathways and diminishing the tumor microenvironment/niches (Beck and Blanpain, 2013; Chen et al., 2013). Since it plays crucial in cancer treatment, CSCs has been an attractive target for the development of most efficient anti-cancer therapies. During past several years increasing number of anti-cancer agents which kill CSCs have been reported, e.g., salinomycin (Gupta et al., 2009), curcumin (Li et al., 2014), thioridazine hydrochloride (Sachlos et al., 2012), sulforaphane (Li et al., 2010), miR-34a (Liu et al., 2011), and miR-130b (Ma et al., 2010). However, the typical rate limiting factors which remain associated with other anti-cancer drugs (e.g., small molecules, peptide-based drugs, and nucleic acid based drugs) are also present with them. For instance, off-target effects, poor water solubility, inconsistent stability, short circulation time, and inefficient distribution along with low therapeutic indexes are most commonly reported limitations (Chen, 2010). Recent advancements in nanomedicine technology have raised the hopes for the development of optimal cancer therapeutics. There are various types of nanocarriers such as liposomes, polymeric micelles, dendrimers, carbon nanotubes and metal nanoparticles, which can overcome the limitations as mentioned earlier (Davis et al., 2008; Rink et al., 2013). The nanoparticle-based drug delivery system own superior pharmacokinetic/pharmacodynamic qualities that make it an excellent method of choice for the cancer management. Nanoparticle offers higher carrier capacities for most of the drugs with improved pharmacokinetic and pharmacodynamic profiles (Sun T. et al., 2014). These properties are carefully controlled through their component type, size and surface characteristics which make them capable of having reduced harmful side-effects (Sun T. et al., 2014). There are few examples of clinically approved nanomedicines (nanocarbon based), e.g., liposomal doxorubicin (Doxil) (Barenholz, 2012), Albumin-bound paclitaxel (Abraxane) (Gordon et al., 2001) and PEG-1 Asparaginase (Oncaspar) (Gordon et al., 2004). There are few more novel and sophisticated nanoparticles with multiple functions which are being evaluated for their various characteristics and would be available in the near future as an advanced version of nanomedicines (Sun Q. et al., 2014). The successful treatment of cancer requires the development of approaches which can efficiently eradicate cancer and much-improved application of this new drug-delivery modality (nanomedicines) (Zhao et al., 2013). There are reports on the development of new regime by using these technological advancements to track the challenges posed by CSCs in the treatment of cancer. For example, a significant reduction in the growth of anchorage-dependent clonogenic growth of CD133+ CSCs in glioblastoma cell-based studies by using Nano-CurcTM (Sign Path Pharmaceuticals, Inc., Pennsylvania, USA; 1.5% curcumin content) (Lim et al., 2011). Recently, Sun et al., demonstrated enhanced anti-cancer activities of doxorubicin and all-trans-retinoic acid through a polymer co-delivery system in human breast cancer mice models (Sun et al., 2015).

Thus, it seems that Nanotechnology-based approaches are going to be the primary tool for developing most effective anti-cancer therapies and an increasing number of CSCs-targeting nanomedicines are being developed and even being evaluated through preclinical studies. For the introduction of these nanomedicines into clinical practice, a large number of detailed experimental and other relevant information is essential and present article focuses on the CSCs related biological processes. The objective of this review is to discuss different nanomedicines targeted toward CSCs and also the limitations associated with their clinical uses. The first section deals with the comprehensive details about the CSCs biology and various therapeutic approaches to tackle them. Whereas, the second part of the article provides a detailed understanding of the different types of Nanocarriers along with their combination of different CSCs management approach.

## CSCs and various therapeutic approaches

**Figure 1:**
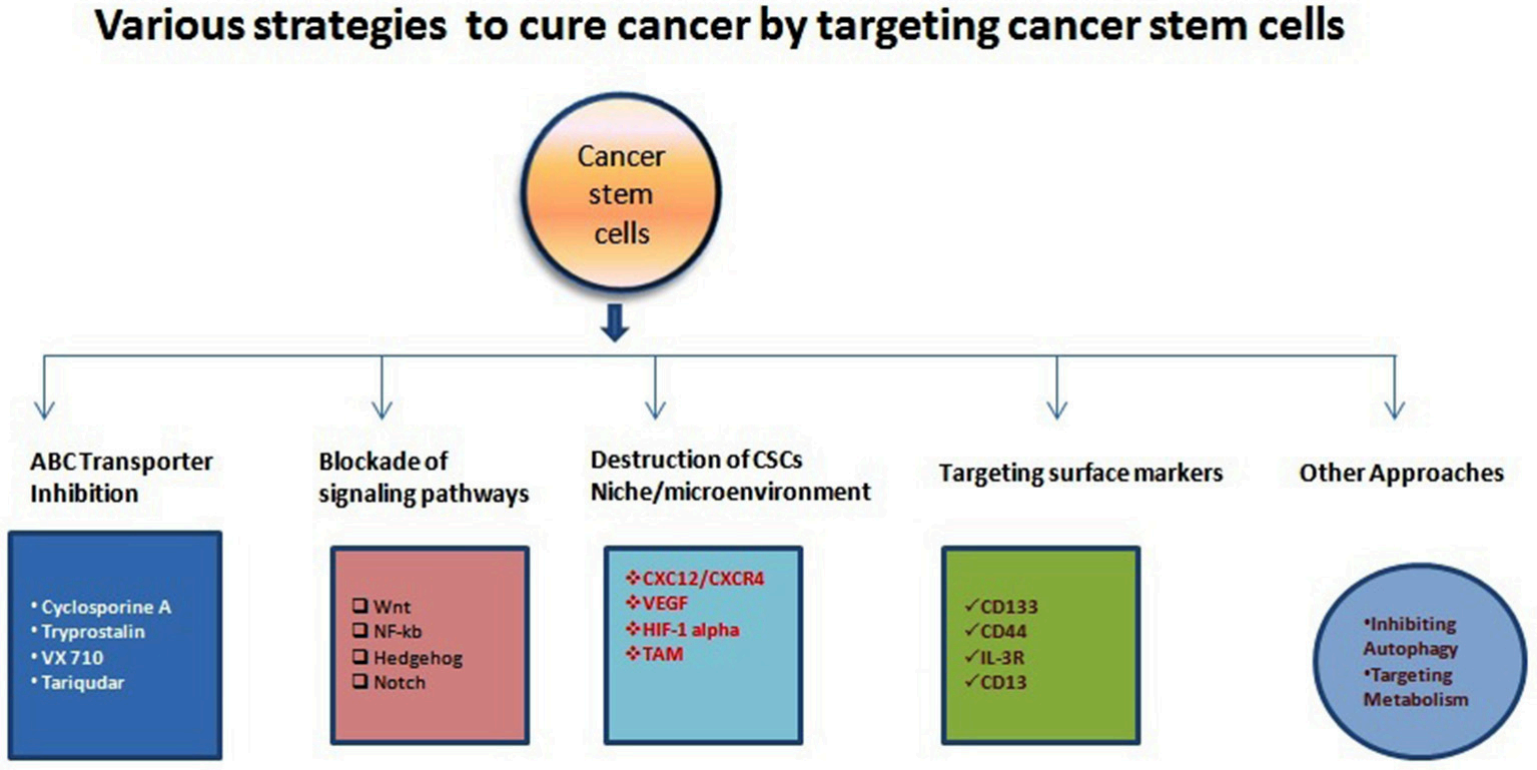
Illustration of various anti-CSCs modalities to cure different types of cancers. There have been accumulating study and clinical report about the various mechanism for targeting CSCs and other cancer cells as indicated above. Various research/clinicians have also demonstrated different molecules or strategies with variable efficiencies.

**Figure 2:**
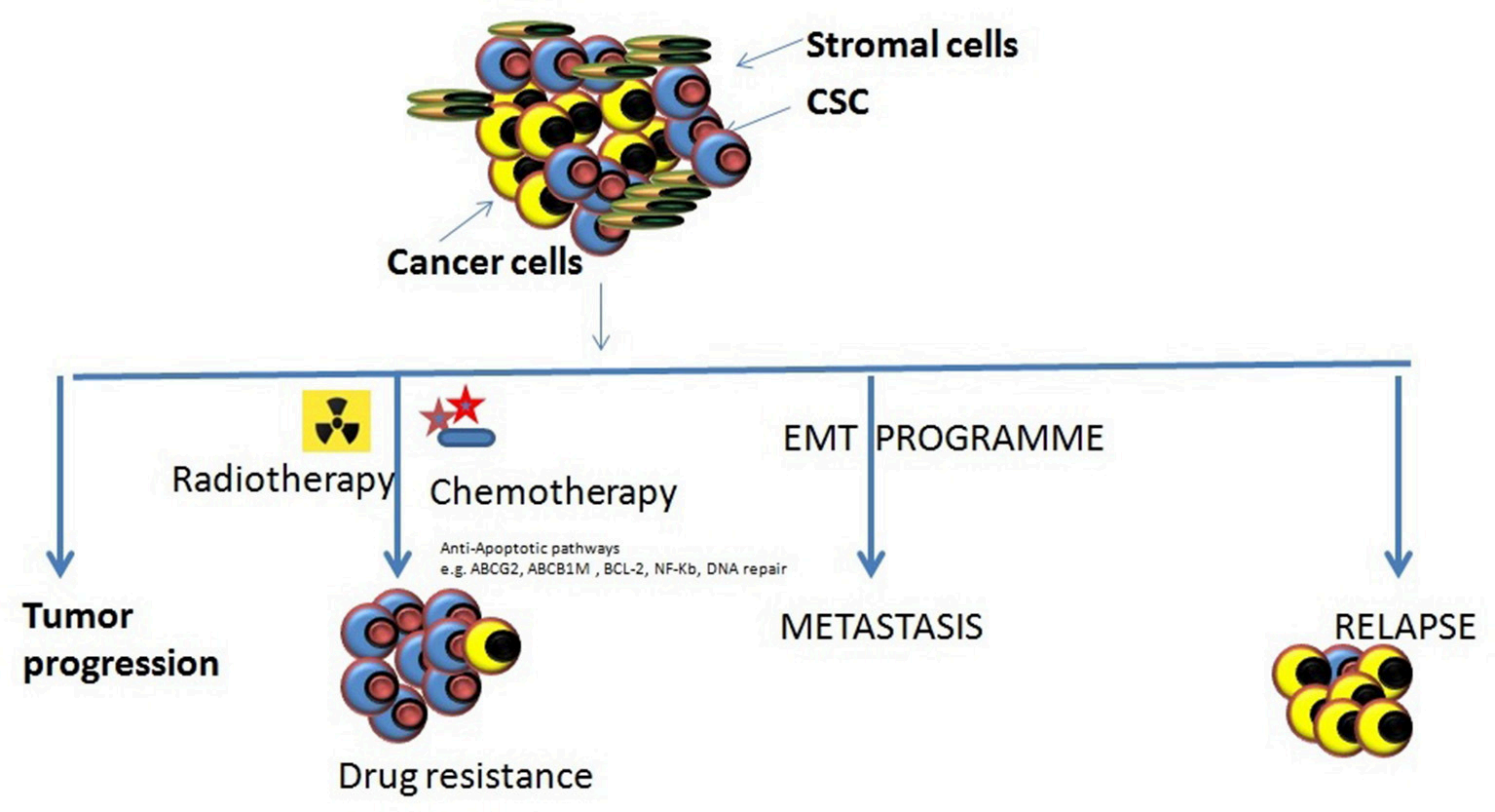
Various roles of CSCs in tumor progression. There are different types of roles which CSCs might play in tumor progression and cause them to become resistant to the most of the conventional therapeutic modalities. Tumor progression: The inherent properties of CSCs to self-renew, proliferate, and differentiation makes them eligible to support tumor progression. Drug resistance: the abilities of CSCs to survive against various cytotoxic insults including chemotherapy/radiotherapy through different mechanism may cause the accumulation of them resulting in enrichment of CSCs within tumors making it harder to cure cancers. Metastasis onset: Acquisition of mesenchymal cell-like features by CSCs it impossible that tumor cell starts migrating to the local and distant locations causing the onset of metastases. Relapse: the remaining CSCs that may survive the anti-tumor treatment remedy can increase their population by proliferating and may result to the relapse after an initial therapeutic success.

In the1800s, *embryonal rest theory* stated the possible relationship between the origin of cancer and stem cells (Sell, 2009). Around 50 years ago various studies started on germinal cell cancer (teratocarcinoma)showing the generation of cancer cells from stem cells, and it proposed a concept that tumors contain different types of stem cells (Sell, 2009). Studies on liver cancer which shown the origin of liver cancer from dedifferentiated mature hepatocytes further strengthen this concept (Sell, 2009). Since then, our understanding of cancer etiology has significantly improved through modern genomic, proteomic, and functional analytical technologies (Hanahan and Weinberg, 2011). Burgeoning information through various cancer studies about the heterogeneity and molecular mechanisms regulating various components of cancer cells has firmly established the existence of cancer stem (-like) cells (CSCs) or Tumor-initiating cells (TICs) (Nguyen et al., 2012). A unique fraction of cells that have self-renewal, differentiation capabilities are further defined by using many specific cell surface markers and various intracellular dyes (e.g., Hoechst, 33342, PKH26) (Oates et al., 2009; Pece et al., 2010). It is a common assumption that CSCs can differentiate into various derivatives that comprise the significant share of tumor tissue. The genesis of CSCs in the solid tumor is not very well understood. It is shown that CSCs may arise from a series of naturally occurring stem cells or some differentiated cell also (Bjerkvig et al., 2005; Bu and Cao, 2012). Reports are indicating crucial role played by epithelial-mesenchymal transition (EMT) programs in generating CSCs in many types of malignancies (Mani et al., 2008; Gupta et al., 2009). The EMT (and reverse process Mesenchymal-Epithelial Transition or MET) play a central role in normal embryogenesis and often gets activated during cancer invasion and metastasis (Hay, 1995; Perez-Pomares and Munoz-Chapuli, 2002). Many transcription factors which have pleiotropic activity have been demonstrated to play a central role in embryogenesis by orchestrating EMTs as reported by several developmental genetic research studies (Briegel, 2006). Further advancements occurred in defining malignant traits, e.g., motility, invasiveness, and resistance to apoptosis in neoplastic cells (Comijn et al., 2001; Oft et al., 2002; Yang et al., 2004; Huber et al., 2005; Savagner et al., 2005; Hartwell et al., 2006; Cheng et al., 2007; Mani et al., 2007; Peinado et al., 2007). Few of these transcription factors might play important roles in wound healing (Savagner et al., 2005). Due to their similarities with normal stem cells, CSCs are believed to be the primary dragging force for tumorigenesis (Medema, 2013). The conventional anticancer treatment like radiotherapy and chemotherapy actually may enrich the CSCs due to their natural longer lifespan and resistance toward the conventional treatment modalities (Dean et al., 2005; Bao et al., 2006a; Woodward et al., 2007). CSCs enrichment has been associated with the ability of tumors to proliferate and disseminate to remote lesions which result in the development of metastasis and also may cause their relapse after initial therapeutic success as reported by studies (Li Y. et al., 2015). Collectively, these characteristics of CSCs make the tumor more resistant toward most of the treatment modalities and a major reason of cancer-related death (Figure 1). It is evident that extensive efforts have been made to develop anti-CSCs therapeutic modalities that can efficiently eradicate CSCs and reduce the risks of metastasis and relapse (Chen et al., 2013). In this direction inhibition of ABC transporters has been very attractive. The ABC transporters notable on the CSC surface and inhibition of these receptors make CSCs more sensitive to the other therapeutic agents and thus improve overall efficiency (Yang et al., 2014). Similarly, surface markers and blockade of signaling pathways could be an effective anti-CSCs therapy (Table 1). Another important strategy may be the demolition or alteration of CSCs microenvironment or niche. Few novel anti-CSCs strategies are also surfacing such as induction of autophagy in CSCs and modulation of abnormal metabolism of CSCs (Figure 2).

**Table 1:** Various cell surface marker used for identification and eradication of CSCs.

**Sr. no**.	**Tumor/Cancer type**	**Phenotype of CSCs markers**
**CSCS MARKERS**		
1	Colon cancer	CD133þ, CD44þ, CD166þ, EpCAMþ, CD24þ, CXCR4þ, CK20þ, CEAþ, LGR5þ
2	Pancreatic	CD133þ, CD44þ, EpCAMþ, CD24þ, ABCG2high
3	Lung cancer	CD133þ, ABCG2high
4	Leukemia	CD34þ, CD38–, HLA-DR–, CD71–, CD90–, CD117–, CD123þ
5	Breast cancer	ESAþ, CD44þ, CD24–/low, Lineage–, ALDH1high
6	Multiple myeloma	CD138–
7	Brain cancer	CD133þ, BCRP1þ, A2B5þ, SSEA1þ
8	Liver cancer	CD133þ, CD49fþ, CD90þ
9	Prostate cancer	CD44þ, α2β1high, CD133þ
10	Head and neck cancer	CD44þ, ALDHþ, YAP1þ, BMI1þ

### Strategies for energy-dependent efflux mechanism to target CSCs

CSCs are believed to occur in tumor tissues, and similar to healthy stem cells CSCs also have a high expression level of ABC transporters (Dean et al., 2005). This ABC transporter expression is correlated with multidrug resistance in CSCs. The ABC transporters are supposed to reduce cellular accumulation of various types of therapeutic agents, and thus CSCs become relatively more resistant to even higher doses of anti-cancer agents (Gottesman et al., 2002). Stem cells are often defined by their ability to get stained very lightly by Hoechst 33342 due to overexpression of a drug efflux protein called BCRP (breast cancer resistance protein) (or ABCG2) which actively pumps out the dye molecules. Therefore, Hoechst 33342 dye efflux method is a common method of choice for the isolation/identification of stem cells as side-population' and tumor cell identification (Goodell et al., 1996; Hirschmann-Jax et al., 2004; Ho et al., 2007; Britton et al., 2012). These findings prompted researchers to develop the inhibitory strategies for the ABCG2 pump, and many such molecules have been evaluated including Fumitremorgin C (Rabindran et al., 2000). Moreover, Tryprostatin (Woehlecke et al., 2003) that are shown to be effective in sensitizing and killing CSCs. There are other molecules which have been associated with drug resistance in CSC, e.g., P-glycoprotein (or ABCB1) and ABCB5 (Nobili et al., 2006; Angelastro and Lame, 2010). PGP is reported as a primary reason for therapy failures in leukemia and solid tumors patients. Similar reports are available ABCB5 which is associated with MDR resistance in human malignant melanomas patients (Frank et al., 2003). It is necessary to notice that blockade of ABCB5 efflux activity by the Monoclonal antibody can sensitize these cells against anti-cancer drug doxorubicin (Frank et al., 2005). These studies indicate the importance of transporters in MDR and targeting these efflux pumps could be an efficient method to control MDR in cancer treatments. Despite the knowledge of MDR for over 30 years, the clinical success in regulating this phenomenon has been insufficient. There have been various strategies proposed and evaluated to overcome MDR such as direct or indirect inhibition of ABC transporters. For example, direct inhibition strategies demonstrated the development of small molecular weight inhibitors or mABs, e.g., Cyclosporine A, VX710, Tariquidar, and indirect inhibition strategies evolved various methods for disrupting cell signaling pathways to inhibit ABC transporter activities (Gottesman et al., 2002). However, all these strategies have been suffering from low inhibition efficiency and unwanted toxic effect which has significantly compromised their clinical use. Therefore, it is evident from existing data that with the presently limited effects all these approached may not be able to overcome the CSC dependent drug resistance and more broad spectrum strategies such as nanocarriers would be important in developing optimal anti-CSCs strategies to explicitly control drug resistance and improve the therapeutic efficacy of any anti-cancer regime.

### Blockade of CSCs-related signaling pathways

CSCs retain their hallmark stem cell-like properties (i.e., self-renewal and differentiation) through the modified or deregulated signaling pathways networks. Studies from several groups have revealed a significant role of Wnt/β-catenin (Takahashi-Yanaga and Kahn, 2010), Hedgehog (Merchant and Matsui, 2010), Notch (Pannuti et al., 2010), Bcl-2, PI3K/Akt, PTEN and NF-κB, in CSCs' self-renewal and differentiation. Moreover, detailed information of these molecules and their related signaling cascade would be essential to develop any effective strategies against CSCs mediated drug resistance or anti-cancer therapy (Chen et al., 2013). Studies have shown significant role of embryonic signaling pathways, e.g., Wnt, Notch, and Hedgehog in maintaining the CSCs population in multiple melanomas (Campbell et al., 2008; Takebe et al., 2011). These raised the possibilities of the development of effective strategies to control drug resistance, and several clinical trials are in progress targeting the Pathways (Zhao et al., 2013). For example, blockade of Notch-I signaling pathway has been demonstrated to reduce the fraction of CD44+CD24 subpopulation and also decreased the instance of brain metastasis of brain cancer (McGowan et al., 2011). Similarly, many pharmaceuticals are being explored, e.g., Wnt signaling inhibitors which are responsible for regulation of CSCs and tumorigenicity, and one such example is the use of mAbs against Wnt signaling cascade is demonstrated to be of significant value in the treatment of colorectal cancer (He et al., 2005). Many independent reports are available on the use of cyclopamine (an SMO signaling element inhibitor) to block the Hedgehog-mediated signaling pathway resulting in inhibition of growth/proliferation, invasion, and metastasis of many malignancies an evident from both the *in vitro* and *in vivo* studies (Karhadkar et al., 2004; Feldmann et al., 2008).

Apoptosis dysregulation is more often defined as a hallmark of carcinogenesis and remains an important determinant of the efficacy of chemotherapeutics to establish malignancy (Brown and Attardi, 2005; Hanahan and Weinberg, 2011). CSCs are reported to use several mechanisms to dysregulate signaling pathways and enhance resistance against most of the chemotherapeutic regimes. BCl-2 family of anti-apoptotic proteins is the most studied and characterized regulators of apoptosis. It would be interesting to notice that BCL-2 (an anti-apoptotic protein) is very well known to regulate repopulation potential and provides protection against apoptotic insults in hematopoietic stem cells (Domen et al., 2000). BCL-2 expression is reported to be significantly high in comparison to healthy cells as demonstrated in an *in vitro* model of blast-crisis CML-derived CSCs and their non-CSCs counterparts (Goff et al., 2013). Whereas, the frequency of breast CSCs is highly reduced by expression of a strongly active pro-apoptotic BIK mutant both in cell lines and patient studies (Lang et al., 2011).

Recently, the relationship between CSCs and nuclear factor kappa B (NF-κB) has been elucidated by several researchers. NF-κB is a transcription factor, and it regulates the expression of numerous genes and mediates various cellular responses such as cytokines, radicals and UV irradiation induced pathways (Baud and Karin, 2009). The constitutive activation of STAT-3/NF-κB signaling cascade and enhanced expression of TAT-3/NF-κB dependent gene has been demonstrated in glioma CSCs (Garner et al., 2013). In similar stream, use of curcumin based approach has been shown to reduce stem cell properties in breast CSCs through modulation of TAT-3/ NF-κB signaling cascade (Chung and Vadgama, 2015).

Additionally, secreted protein, e.g., cytokines have also had a significant impact on CSCs survival, and some studies have shown that strategies against these secretory proteins may also be of importance in counseling resistance and sensitize the CSCs against chemotherapeutics. This is evident from the reports showing significant sensitization of colorectal CSCs in the treatment of freshly isolated CD133+ cells with anti-IL-4Ra antagonist or anti IL-4 neutralizing antibodies against standard chemotherapeutic drugs (Todaro et al., 2007). Similarly, inhibition of CXCR1 through small molecular inhibitor has been demonstrated to diminish residual breast CSCs population following docetaxel treatment *in vivo* (Ginestier et al., 2010). THE IL-8 receptor CXCR1 is strongly expressed in breast cancer CSCs (Ginestier et al., 2010).

### Regulation through tumor microenvironment/niche targeting

Studies on the tumor microenvironment have revealed their similarity with stem cells and CSCs niche can be defined as an anatomically distinct region which consists of various types of cells, e.g., mesenchymal cells vascular cells and inflammatory cells along with diffusible molecules and extracellular matrix proteins (Plaks et al., 2015). These niches maintain the CSCs' stem cell properties. These niches preserve the various characteristic of CSCs, e.g., phenotype, plasticity, and also protect them against drug-induced apoptosis and facilitate their metastatic potential (Oskarsson et al., 2014; Ye et al., 2014). Thus, targeting CSC niche could be a powerful tool in controlling the tumor progression and their treatment. There have been few promising attempts to target CSCs niche. It is reported that tumor angiogenesis may have a direct relationship with the survival and drug resistance of CSCs. Further, CSC in the vascular niche can comprise an autocrine loop that involves VEGF-mediated promotion of CSCs activities which are regulated through the formation of microvasculature and intrinsic self-renewal pathways (Bao et al., 2006b; Beck et al., 2011). Thus, inhibition of VEGF activities can normalize tumor vasculature, and that can result in the disruption of CSCs microenvironment/niche causing reduced tumor growth (Vermeulen et al., 2010). Another useful modality to disrupt CSC niche may be targeting tumor hypoxia. For instance, HIF-1α and HIF-2α (regulators of the cell cycle through c-Myc) may be targeted to control the growth of quiescent, drug-resistant tumor cells in glioma patients (Li Z. et al., 2009). An alternative approach may be targeting against tumor-associated stromal cells (e.g., myofibroblast and tumor-associated macrophage) that play a significant role in homeostasis regulation in various tumors, and their inhibition may diminish CSCs growth also (Raaijmakers et al., 2010; Vermeulen et al., 2010).

### Targeting against cell surface markers

Cell surface marker holds the key for generating specific strategies against CSCs, and thus considerable efforts have been put in the development of more precise therapeutic regimes in various types of tumors. For example, use of an activating anti CD44 mAbs in a NOD/SCID-human AML transplant mice model shown a significant reduction in the leukemic cell population (Jin et al., 2006). Specific eradication of leukemic stem cells in human have been demonstrated by targeting other surface molecules also which are differentially expressed in normal and CSCs such as IL-3R (Jin et al., 2009) and TIM-3 (Kikushige et al., 2010). Similar reports are available from mice studies where treatment with antibodies against IL-3R and TIM-3 has managed to diminish leukemia cells. The self-renewal of tumor cells and the tumor-initiating ability of the dormant CSCs were significantly suppressed by targeting CD13 with the treatment of anti-CD13 which is a specific liver CSCs marker (Haraguchi et al., 2010). The significant anti-cancer therapeutic potential is revealed by studies in which CD133 was targeted in lung cancer (Bertolini et al., 2009), liver cancer (Rountree et al., 2009), and glioblastoma (Brescia et al., 2013). Similar reports are available on the inhibition of self-renewal and tumorigenic capacity of neurosphere cells through down-regulation of CD133 gene excretion by short hairpin RNA (shRNA-mediated method; Brescia et al., 2013).

### Various other approaches

#### Targeting autophagy signaling pathway in CSCs

Autophagy may be defined as an evolutionarily conserved mechanism through which cells responds to the different types of environmental stress such as starving, exposure to radiation, hypoxia, and chemotherapeutic agents (Choi et al., 2013). Inhibition of autophagy has been demonstrated to enhance the sensitization of cancer cells (Yousefi and Simon, 2009; Sui et al., 2013). However, similar effects on CSCs are not very much clear and remain controversial. Recent studies have shown that inhibition of autophagy through knock-down of autophagy-associated genes or direct autophagy inhibitors may lead to reduced stem cell self-renewal, differentiation, and ability to resist various types of stresses which may result in reduced CSCs population and enhanced sensitivity (Cufi et al., 2011; Zhuang et al., 2011; Maycotte et al., 2015). The combination of autophagy inhibition strategies along with standard anti-cancer therapeutic agent may be more efficient in eradicating CSCs completely and curing cancers (Zhou et al., 2007; Hirsch et al., 2009; Balic et al., 2014).

#### Regulation of CSCs' metabolism

Cancer cells are defined as having deregulated proliferation due to uncontrolled metabolic activities (Ward and Thompson, 2012). CSCs are also reported to exhibit distinctive metabolic characteristics (Menendez et al., 2013). For example, tumor cells (especially brain tumor cells) express high levels of Glucose Transporter 3 (GLUT3). Moreover, it is known down through shRNA can lead to significant drop in the frequency of brain tumor stem cells (*in vitro*) and glioblastoma formation (*in vivo*) (Flavahan et al., 2013). Furthermore, basal-like breast CSCs are reported to have distinct glucose, and mevalonate metabolism and metabolic drug metformin have been shown to exhibit anti-CSCs properties and also can improve the efficacy of chemotherapeutics (Ginestier et al., 2012; Hirsch et al., 2013; Wurth et al., 2013).

## Nanomedicine as an effective tool against CSCs

The biodegradable and biocompatible nano molecules or nanocarriers have been reported to deliver a broad range of therapeutic molecules (Peer et al., 2007). This accumulating list of therapeutic molecules includes hydrophobic/hydrophilic drugs (Lei et al., 2013; Li et al., 2014; Zhu et al., 2014), various types of peptides and proteins (Grenha et al., 2005), imaging probes (Park et al., 2009), antibodies (McCarron et al., 2008), nucleic acids (Tan et al., 2014), and even multiple drugs simultaneously (Patil et al., 2010). Nanocarriers offer a number of benefits to the active drug molecules by protecting it from harsh biological conditions and thus enhance their pharmacokinetic and pharmacodynamic profiles significantly (Hamaguchi et al., 2005; Yuan et al., 2012). For example, PEGylated nanocarriers (can evade reticuloendothelial system) are reported to have prolonged circulation time and significantly less accumulation in the healthy tissues in comparison to non-PEGylated nanoparticles (Jokerst et al., 2011). Similarly, stimuli-responsive nanocarriers are developed with the capacity to respond to the various external stimuli such as pH, temperature and light and thus provide a control over the release of drugs/therapeutic molecules to the target sites only (Soppimath et al., 2005; Li Y. Y. et al., 2009; Du et al., 2011; Gao et al., 2011; Lee et al., 2011; Li et al., 2011). The nanoscale size of these carrier molecules provides them capabilities to get accumulated in the tumors because of enhanced permeability and retention (EPR) effect. Further, nanoparticles equipped with specific targeting moieties, e.g., folate antibodies and aptamers, etc. can become more precise in delivering their cargo. There have been sufficient advancements in describing various drugs, protein, and gene delivery using nanoparticles to target CSCs (Tables 2–**5**) through conventional therapeutics by using different resistance mechanism as discussed above. Nanomedicine, as discussed below, offers various advantages over the conventional therapies, and the importance of nanomedicine.

**Table 2:** CSC specific small therapeutic agents and their delivery.

**Sr. no**.	**Anti-CSCs strategies**	**Drugs/Therapeutic agent**	**Nanocarriers based delivery system used for the therapeutic agents**	**CSC source and specific marker**	**References**
1.	Selective inhibition of Human multiple CSCs	Phenformin	Polymeric micelles using PEG-b-PAC and PEG-b-PUC 102 nm particle	Lung cancer, H460 cells, CD133+ human lung cancer mouse model	Krishnamurthy et al., 2014
2.	Selective inhibition of CSCs	Salinomycin	SAL-SWNTCHI-HA complexes; self-assembled nanoparticles from iTEP; nanogel-drug conjugates based on membranotropic CHA	Gastric cancer, AGS cells, CD44+; murine breast cancer, 4T1 cells, CD44+CD24–; breast cancer, MDA-MB-231 cells, CD44+	Wei et al., 2013; Yao et al., 2014; Zhao et al., 2014
3.	Enhanced accumulation of drug molecules in CSCs	Oxaliplatin	CSO-SA polymeric micelles	Colorectal cancer, HT29 and SW620 cells, CD133+/CD24+	Wang et al., 2011
4.	Suppression of IGF and STAT3; blockage of Hedgehog pathway	Curcumin	NanoCurc™; stearic acid-g-chitosan oligosaccharide (CSO-SA) polymeric micelles	Brain cancer, DAOY cells, etc., CD133+; colorectal cancer, patient-derived cells, CD133+/CD24+	Lim et al., 2011; Wang K. et al., 2012
5.	Inhibition Hedgehog (Hh) signaling pathway	Cyclopamine	HPMA-based delivery system	Prostate cancer, RC-92a/hTERT cells, CD133+/integrinα2β1hi	Zhou et al., 2012
6.	Selective inhibition of basal-like triple negative breast cancer CSCs	Bortezomib	Poly(ethylene glycol)-b-poly(d, l-lactide) (PEG-PLA) nanoparticles	Breast cancer, SUM159, and HCC1973 cells, ALDH+	Shen et al., 2015
7.	Increased accumulation of chemical drug within CSCs	Doxorubicin	Endosomal pH-responsive DOX-Hyd@AuNPs	Breast cancer, MDA-MB-231 cells, etc., CD44+CD24−ALDH+	Sun T. M. et al., 2014
8.	Increased accumulation of chemical drug within CSCs	Epirubicin	Nanodiamond drug complex	Murine hepatocellular carcinoma, LT2-MYC cells, MYC+	Wang H. X. et al., 2014

### Bioavailability of CSCs specific drugs

Despite the fact that significant advancements in the knowledge about CSCs various problem exist in tackling CSCs due to limitations associated with different anti-CSCs agents which suffer from problems of solubility, degradation, early clearance, limited cellular uptake, and cytotoxicity (Minko, 2004; Sahay et al., 2010).

Here, nanoparticles offer high-capacity carrier capabilities for chemotherapeutic/nucleic acid drug molecules and exhibit greater bioavailability and activity. The largest porosity of the vasculature and impaired lymphatic drainage system in tumors facilitates the passive accumulation of nanoparticles and thus drugs molecules which are attached to them (Gao et al., 2012). Alternatively, nanoparticles may be connected with high-affinity molecules against specific receptors which are exclusively expressed on the tumor cells or CSCs and thus may enhance delivery specifically to these sites (Xia, 2014). Most studies report that conventional therapeutic agents have limited access to the CSCs due to their hypoxic microenvironment and distant location away from the vasculature that retards the efficacy of anti-CSCs drugs (Mohyeldin et al., 2010). However, rationally designed nanoparticles may overcome this limitation and may penetrate up to a deeper location and kill CSCs through anti-CSCs drugs molecules.

### Drug resistance of CSCs vs. nanoparticles

Rationally designed nanoparticles have been reported to be effective against the MDR predominantly ATP-driven transporters which remain an axial factor in most of the MDR and the intractable obstacle of CSCs (Markman et al., 2013). Studies reported so far on the use of chemotherapeutic agents, which are either conjugated to nanoparticles or encapsulated by nanoparticles, are not recognized as a solid substrate by ABC transporters system and the can stay for longer periods. Additional advantages are provided through cell penetrating peptide- or targeting moiety-modified nanoparticles which provide protection against receptor-mediated and energy drove endocytosis or macropinocytosis that significantly increase intracellular accumulation of drugs (Livney and Assaraf, 2013). Thus, specifically designed nanoparticles may greatly enhance intracellular accumulation/concentration of CSCs targeting agents that would improve their cytotoxic effects.

### Reduced off-target effects

The field of identification of new CSC marker (biomarkers) and signaling pathways facilitated by genome-wide screening methodologies are tremendously grown up in recent years. Unfortunately, most of the biomarkers are also shared by normal stem/progenitor cells, and drug targeted to these biomarkers may also lead to acute and irreversible damage to the normal tissues causing organ failure, etc. (Hu and Fu, 2012). More comprehensive strategies would be essential to avoid these shortcomings of the existing approaches. For designing specific therapeutic agents, it would be essential to delineate among the CSCs and other healthy stem cells so that only CSCs are killed while other normal stem cells remain unaffected. Also, the newer developed strategies should have the potential to realize differential delivery of CSCs-killing drugs. Since tumors have an impaired vasculature/lymphatic drainage system, the drug-loaded nanoparticles are accumulated in a comparatively higher concentration of tumor cells that prevent damage to the normal stem cells to a limited extent. Whereas, free chemical or biomolecules are distributed equally among tumor and healthy stem cells that may damage to the normal stem cells causing unwanted organ failure or other adverse symptoms (Davis et al., 2008).

## Nanomedicines: few examples

**Figure 3:**
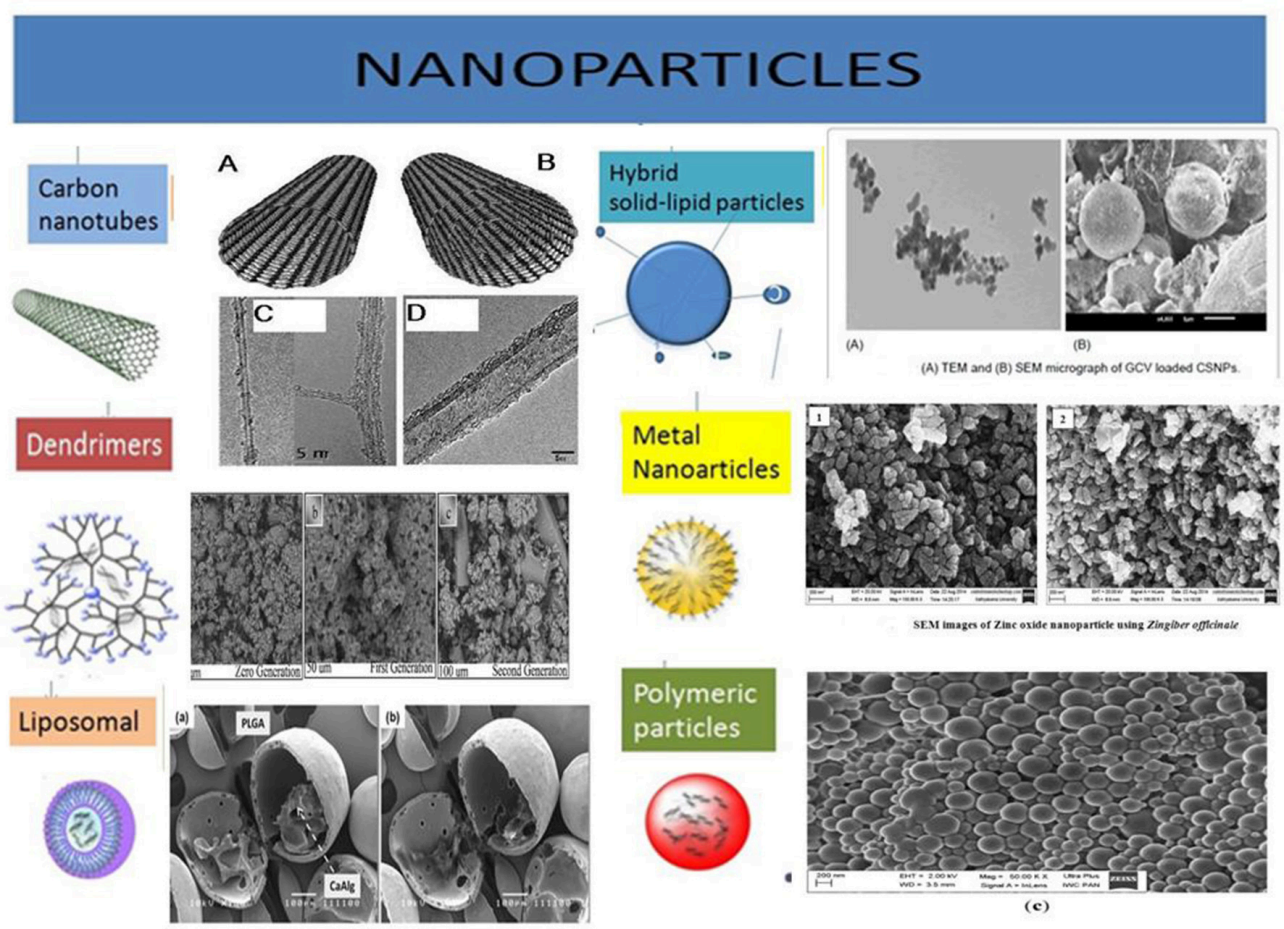
Illustration of various types of nanoparticles being explored for their efficiency to carry desired anti-CSCs/anti-cancer drug molecules. These nanocarriers are often equipped with targeting moieties, e.g., antibodies, antigen, etc. the different types of nanoparticles are developed from many types of biomaterial, e.g., lipids, metals, carbon, polymeric substances, etc. Acknowledgment: The various nanoparticles SEM/TEM figures are taken from the previously published work with prior permission/OR accessible under open access. Carbon Nanotubes: Eatemadi et al. (2014) (Open Access). Dendrimers: Abd-El-Aziz et al. (2016) (Permission granted by author). Liposomal: Lim et al. (2013) (Permission granted by author). Hybrid solid-liquid particles: Patel et al. (2016) (Open access). Polymeric particles: Halayqa and Domańska (2014) (Open Access). Metal nanoparticles: Raj and Jayalakshmy (2015).

Burgeoning knowledge of CSCs biology and recent developments in the nanotechnologies has fed the development of a vast array of anti-CSCs targeting systems. Here, is the summary of nanomedicines reported so far and which have been categorized on the basis of their introduction and evaluation in for desired effects, type of cargo and modifications of the nanoparticles (Figure 3).

### Nanotechnology may improve CSCs specific therapeutic agents' delivery

CSCs specific therapeutic agents' physiological and physicochemical characteristics can be analyzed similarly to the traditional anti-cancer drugs *in vivo*. It is well established that nanocarriers might be a useful tool in qualifying therapeutic agents (Table 2).

The antibiotic salinomycin (SAL) could be a good example which has been identified through high-throughput screening methods as a potent anti-CSCs agent (Gupta et al., 2009) but exhibit poor water solubility and high toxicity, which makes it unsuitable for clinical uses. Nanocarrier offers a solution to this problem, for example, SAL-SWNTCHI-HA (a gastric anti-CSCs targeted drug delivery system) has been demonstrated to acquire high bioavailability and limited toxicity of SAL. This combination was shown to downregulate self-renewal capability of the CD44+ cell population and also reduced the formation of mammosphere by CSCs (Yao et al., 2014). Another great example could be Curcumin which has high anti-cancerous potential, but due to hydrophobicity, poor stability, and pharmacokinetic characteristics *in vivo* applications are limited.

Nanotechnology offers better utilization of these therapeutic molecules as the development of curcumin nanoparticle encapsulated in polymeric micelles could show increased accumulation of curcumin in cancer cells resulting in effective eradication of CSCs (CD44+CD24− subpopulation) both *in vitro* and *in vivo* studies in colorectal cancer studies (Wang K. et al., 2012; Li and Zhang, 2014). Similarly, nanoparticles carrying embryonic signaling inhibitory agents in CSCs have also been reported but with limited clinical utility due to their poor solubility and diverse side effects. For example, N-(2-hydroxypropyl) meth acrylamide (HPMA) conjugate of cyclopamine (a Hedgehog pathway inhibitor) has been reported showing potential to eradicate CD133+ cells in the human prosthetic cancer epithelial cell line (RC-92a/hTERT) and exhibit relatively decreased systemic cytotoxicity (Zhou et al., 2012).

Similarly, nanocarriers can improve the efficacy of conventional chemotherapeutic agents by delivering them to CSCs in a more efficient manner and may enhance their anti-cancer activities. For instance, tethering of Doxorubicin with the gold nanoparticles via a poly (ethylene glycol) spacer and an acid-labile hydrazone bond (DOX-Hyd@AuNPs) enhanced delivery of the drug molecules to breast CSCs and reduced drug resistance in these cells through the inhibition of their efflux by PGP. DOX-Hyd@AuNPs exhibited inhibition of CSCs enrichment and tumor growth during or after the treatment (Sun T. M. et al., 2014). Another excellent example is shown use of stearic acid-g-chitosan oligosaccharide (CSO-SA) polymeric micelles to deliver oxaliplatin exhibiting significantly enhanced internalization of OXA-loaded CSO-SA micelles in colorectal CSCs causing reversal in chemoresistance abilities of CD133+/CD24+ CSC subpopulations and enhanced cytotoxicity both *in vivo* and *in vitro* studies (Wang et al., 2011).

### Nucleic acids dependent anti-CSCs drugs and nanomedicines

Nanocarriers are helpful in increasing the solubility, stability, and bioavailability of various macromolecular drug agents (Kalota et al., 2004). One of the important nucleic acid family playing crucial roles in post-transcriptional regulations is small RNA molecules, e.g., micro RNA (miRNA) that regulates various cellular functions. miRNA have recently been evaluated for their abilities to provide a prognostic marker for CSCs and anti-cancer agent in different tumors (Ma et al., 2010; Liu et al., 2011). RNA interference (RNAi) possessing capabilities of specifically regulating targeted gene offer potential treatments for a broad range of diseases including cancers (Pai et al., 2006). Specifically designed RNAi to suppress the cancer-promoting key molecules may be an important method for their treatment. For instance, suppression of OCT-4 gene through RNAi technique could result in the induction of apoptosis in CSCs breast cancer and lung carcinoma cells (Hu et al., 2008). However, poor cellular intake, off-target activity, sensitivity to nucleases, and risks of systemic toxicity may limit their therapeutic potential (Muthiah et al., 2013). Similar to other anti-cancer drugs nucleic acids related therapeutic agents require the development of strategies for their protection from nuclease-driven degradation and enhancement of tissue-specific penetration and accumulation causing improved anti-cancer activity (Wu et al., 2011).

A significant reduction in the tumorigenicity of CD133+ improved differentiation of them into healthy cells (CD133- non-CSCs) on using polyurethane-short branch polyethyleneimine (PU-PEI) as a nanocarrier for delivering miRNA-145 into glioblastoma cells. The delivery of PU-PEI/miR-145 nanoparticles in these studies effectively blocked the expression of drug-resistance associated genes and thus improved the sensitivity of drug-resistant CSCs against other anti-cancer agents (Yang et al., 2012). Similar studies reported using cationic lipid nanoparticles for the delivery of pre-miRNA-107 (regulator of proliferation and survival related genes) as an effective tumor suppressing agents in model head and neck squamous cell carcinoma (HNSCC) showing decrease tumor sphere-forming capabilities through the down-regulation of stem cell transcription factors (Piao et al., 2012). Delivery of miRNA-34a through solid lipid nanoparticles was also demonstrated to downregulate differentiation and metastasis of CSC by direct repression of CSCs marker CD44 in lung cancer cells (Shi et al., 2013). Similar studies with siRNA indicated enormous therapeutic potential but limited due to several obstacles that can be overcome by using nanoparticles based delivery systems (Williford et al., 2014). In an orthotopic lung cancer mouse model, the cationic lipid nanoparticles carrying shAnxA2 (CLG-shAnxA2) showed a significant reduction in drug resistance phenomenon of CSC through suppression of AnxA2 (Andey et al., 2014). An effective anti-cancer strategy could be directed targeting GLUT3 by siRNA-based nanomedicines which block self-renewal and bulk glioma cells in glucose-restricted tumor niche. Further, these GLUT3 blocking nanoparticles could also diminish tumor growth in a U87MG xenograft model (Xu et al., 2015).

In addition to these strategies, another unusual approach is silencing the genes regulating drug efflux transporters and thereby increasing the drug sensitivity of CSCs against anti-cancer agents. For example, employing MDR-1 silencing siRNA-based PEI-Lipid cross-linked (1:16 ratio) nanocarriers shown increased sensitivity of CD133+ human colon cancer cells to the paclitaxel (Liu et al., 2009; Table 3).

**Table 3:** CSCs-specific nucleic acid drugs and their implications.

**Sr. no**.	**Source and marker of CSC**	**Therapeutic agent and drug delivery system**	**Mechanism of CSC depletion**	**References**
1.	HNSCC, patient-derived cells, ALDH1+/CD44+	*siEZH2/siOct4*	Repression of EMT program	Lo et al., 2010
		PU-PEI		
2.	Gastric cancer, BGC823 cells, CD44+	*microRNA-200c*	Regulation of self-renewal, invasiveness and differentiation	Cui et al., 2014
		Gelatinase-stimuli PEG-Pep-PCL nanoparticle		
3.	Melanoma, B16F10 cells, CD44+	*microRNA-34a*	Regulation of CSC differentiation and metastasis	Shi et al., 2013
		Solid lipid nanoparticles (SLNs)		
4.	Non-small cell lung cancer, H1650 cells, side population	*shAnxA2*	Inhibition of resistant phenotype of SP cells	Andey et al., 2014
		Liposomal (cationic ligand-guided, CLG)		
5.	Glioblastomas, U87MG and U251 cells, CD133+	*siGLUT3*	Metabolism of glioma SC targeting	Xu et al., 2015
		Cationic lipid-assisted PEG-b-PLA nanoparticle		
6.	Colon cancer, CHOK1 cells, CD133+	*siMDR1*	Silencing of multidrug resistance gene	Liu et al., 2009
		Nanoparticle consisting of PEI(1200), polyethylene glycol and lipid-based cross linking moiety		
7.	HNSCC, CAL27 cell, etc., Nanog, Oct3/4, and Sox2	*Pre-miR-107*	miR-107 mediated suppression of tumor growth	Piao et al., 2012
		Cationic lipid nanoparticles		
8.	Acute myeloid leukemia, KG-1 and KG-1a cells, CD34+	*siCD44*	Inhibition of LSC interactions with microenvironment	Gul-Uludag et al., 2014
		Nanoparticle consisting of PEI2-caprylic acid and PEI2-linoleic acid		
9.	Glioblastomas, patient-derived cells, CD133+; lung cancer, patient-derived cells, CD133+	*microRNA-145*	Regulation of stem cell-like genes; Inhibition of the EMT program and metastatic ability.	Chiou et al., 2012; Yang et al., 2012
		Polyurethane-short branch polyethyleneimine (PU-PEI) as delivery vehicle		

### Anti-CSCs specific other combinational delivery approaches

Since tumors consist of heterogeneous tissue and various types of cells have a difference in their abilities to respond to these anti-cancer agents. Further, CSCs are capable of showing the reversible transition between stem cell and non-stem cell state (Meacham and Morrison, 2013) which increase the complexity of determining the most suitable anti-CSC strategies. Ther are a clear indication of the fact that eradication of CSCs only may not be sufficient to suppress tumor progression entirely. Residual differentiated tumor cells can be converted into CSCs and may sustain tumor growth even after the complete eradication of CSCs (Bu and Cao, 2012). Thus, a combined approach which can tackle both bulk non-CSCS and reminiscent rare CSCs would be of more importance providing better therapeutic effects (Table 4).

**Table 4:** Various strategies of combinational delivery of chemotherapeutics and CSC-specific agents.

**S. no**.	**CSC source and marker**	**Drug delivery system and combined chemotherapeutics**	**Mechanism of CSCs depletion**	**References**
1	Multiple myelomaJJN3 cells, CD138−CD34–	*Cremophor® EL*	Increased efficacy of conventional chemotherapy	Yang et al., 2014
		Anti-ABCG2 and paclitaxel		
2	Breast cancer, MCF-7 cells, CD44+/CD24–	*PEG-b-PCL polymeric micelles*	Simultaneous killing of CSCs and non-CSCs	Zhang Y. et al., 2012
		Salinomycin and paclitaxel		
3	Glioblastoma, U87 cells, etc., CD133+ and SSEA-1+	*Liposome*	Sensitization of glioblastoma to chemotherapy	Kim et al., 2014a
		Wtp53 plasmid DNA and Temozolomide		
4	Breast cancer, BT474 cells, etc., CD44+/CD24–	*Nanoparticle consisting of PEG-PAC and PEG-PUC*	Simultaneous killing of CSCs and non-CSCs	Ke et al., 2014
		Thioridazine and doxorubicin		
5	Colon cancer, HT-29 cells, CD133+	*Biodegradable lipid nano complex*	Sensitization of CSCs to chemotherapy	Liu et al., 2009
		siMDR1 and paclitaxel		
6.	Breast cancer, MDA-MB-231 cells, ALDH+	*PEG-PLA nanoparticle*	Increased therapeutic response of CSCs	Li S. Y. et al., 2015
		Decitabine and doxorubicin		
7.	Breast cancer, MCF-7 and MDA-MB-231 cells, CD44+/CD24–	*Liposome*	Simultaneous killing of CSCs and non-CSCs	Liu et al., 2008
		Vinorelbine and parthenolide		
8.	Breast cancer, MCF-7 cells, CD44+/CD24–	*Hyaluronan modified mesoporous silica nanoparticle*	Simultaneous killing of CSCs and non-CSCs	Wang et al., 2013
		8-hydroxyquinoline and docetaxel		
9.	Gastric cancer, BGC-823 cells, CD44+	*Elatinases-stimuli nanoparticles*	miR-200c mediated inhibition of CSCs and restoration of drug sensitivity	Liu Q. et al., 2013
		miR-200c and docetaxel		
10.	Breast cancer, MDA-MB-231 cells, ALDH+	*PEG-PLA nanoparticle*	Differentiation of CSCs and increase of chemosensitivity	Sun et al., 2015
		All-trans-retinoic acid and doxorubicin		
11.	Prostate cancer, PC-3 cells, etc., CD133+	*HPMA copolymers*	Simultaneous killing of CSCs and non-CSCs	Zhou et al., 2013
		Cyclopamine and docetaxel		

One notable example of this approach could be the demonstrated by using a combination of paclitaxel [(octreotide (Oct)-modified paclitaxel (PTX)-loaded PEG-b-PCL polymeric micelles (Oct-M-PTX)] and salinomycin [(SAL)-loaded PEG-b-PCL polymeric micelles (M-SAL)] based nanomedicine molecules. The combined effects of paclitaxel (against bulk cancer cells) and salinomycin (anti-CSCs effect) boosted the anti-cancer implications of these drugs *in vivo* and *in vitro* (Zhang Y. et al., 2012). The study of combined effects of HPMA copolymer-cyclopamine conjugate (P-CYP) (anti-CSCs effects), and HPMA copolymer-cyclopamine conjugate (P-CYP) (effective against bulk tumor cells) showed significant enhancement in their tumor growth inhibiting activities (Zhou et al., 2013). Similar intense anti-CSCs activity and anti-bulk tumor cells capability demonstrated by the combined use of other therapeutic molecules. For instance, doxorubicin (DOX) [via acid-functionalised poly(carbonate) (PAC) and poly(ethylene glycol) diblock copolymer (PEG-PAC)] and thioridazine (THZ) [via urea-functionalised poly(carbonate) (PUC) and PEG diblock copolymer (PEG-PUC)] in BT-474 xenografts studies shown stronger effects (Ke et al., 2014). Nanoparticles can function as a carrier for simultaneous delivery of multiple anticancer agents to exhibit better anti-cancer efficacy. For example, simultaneous encapsulation of all-trans retinoic acid (ATRA) (differentiation inducer of CSCs) and Dox and their systemic delivery for breast cancer treatment have been demonstrated to significantly downregulate both the CSCs resistance and tumor growth (Sun et al., 2015). Similar to conventional anti-tumor agents, the nucleic acid drugs are also reported to be co-delivered through nanocarriers based delivery system (e.g., miR-200c, effective anti-CSCs, and docetaxel) with spectacular effects on CSCs proliferation and decrease the migration/invasion and expression of cadherin/CD44 surface adhesion molecules (Shimono et al., 2009). Further, the systemic administration of miR-200c/DOC combined nano-medicine resulted in prolonged retention and more efficient anti-tumor activities in xenograft gastric cancer mice models (Liu Q. et al., 2013). In another approach, the chemotherapeutic agent loaded nanoparticles combined with anti-ABC transporter antibodies and employed on CD130-CD34-CSCs in multiple myelomas showed the enhanced efficiency of PTX and reduced CSCs proliferation and migration (Yang et al., 2014).

### Targeted therapies of anti-CSCs drugs

Anticancer antibodies are useful in inducing tumor regression through their anti-CSCs potency as shown in clinical reports (Vinogradov and Wei, 2012). Apart from that, antibodies are helpful in directing the therapeutic agents to the CSCs, e.g., antibody-drug conjugates antibodies conjugated nanoparticles which can recognize various specific cell surface antigens on CSCs. This approach was used by few researchers to conjugate anti-CD133mANs with the polymeric PLGA nanoparticles bearing paclitaxel drug molecules (CD133BNPs), and these conjugated particles were demonstrated to have significant reducing effects on the number of mammospheres and colonies formation through *in vitro* assays. Comparison of CD133NPs with free drug molecules and non-antibody conjugated nanoparticles in MDA-MB-231 xenograft mice model has shown a significantly higher adverse effect on the CSCS population and improved therapeutic efficacy (Swaminathan et al., 2013). Similarly, the extracellular glycosaminoglycan matrix protein Hyaluronic acid recognizes CD44 which is overexpressed in most of the CSCs (Wei et al., 2013). This specific binding capability of HA is useful in developing CD44-targeted HA-based self-assembling nanosystems for siRNA delivery. This nanoparticle was reported acquiring higher delivery rate and more efficient gene silencing activities in CD44+ (overexpressing) drug resistant tumor cells (Ganesh et al., 2013). Similar molecule chitosan (chemically resembles HA) was useful for targeted delivery of nano doxorubicin or nDox (Dox molecules encapsulated in PluronicF127 nanoparticles) (Rao et al., 2015). nDOX was demonstrated to exhibit a higher cytotoxic effect in comparison to free doxorubicin in CD44+ CSCs residing in 3D mammary spheroids. Further, nDox showed the significant negative effect on the tumor size progression in orthotopic xenograft tumor model. Similar reports are available where anti-CD44 antibodies conjugated with liposomal nanoparticles carrying doxorubicin drug molecules can selectively target CD44+ CSCs in hepatocellular carcinomas and limiting the side effects of conventional chemotherapy (Wang L. et al., 2012).

Epidermal growth factor receptor-2 (EGFR-2) and transferrin receptor (TfR) is also useful biomarkers for targeting both cancer cells, and CSCs and delivery of nanoparticles carrying drug molecules specifically to these cells would be an intelligent approach to eradicating them simultaneously. Both the CSCs and non-CSCs are reported to express TfR. There are reports from both *in vitro* and *in vivo* studies by using particles which can recognize TfR and thus deliver desired anti-CSCs agents [(TfR-targeting nano-complex (Scl)-carrying wtp53 gene)] to them in mouse model studies (Table 5).

**Table 5:** Various types of targeted drug delivery systems for CSC therapy and their potential applications.

**Sr. no**.	**CSCs source and marker**	**Ligand/Receptor**	**Therapeutic agents and drug delivery system**		**References**
1.	Breast cancer, MDA-MB-231 cells, CD133+	Anti-CD133 antibody/CD133	Paclitaxel	Nanoparticles formulated using PLGA polymer	Swaminathan et al., 2013
2.	Non-small cell lung cancer, A549 cells, CD44+	Hyaluronic acid/CD44	SSB/PLK1 siRNA	HA-PEI/PEG nanosystems	Ganesh et al., 2013
3.	Breast cancer, MCF-7 cells, CD44+	Chitosan/CD44	Doxorubicin	Pluronic F127-Chitosan nanoparticles	Rao et al., 2015
4.	Hepatocellular carcinoma, HepG2 cells, CD44+	Anti-CD44 antibody/CD44	Doxorubicin	Liposomal nanoparticle	Wang L. et al., 2012
5.	Colorectal cancer, etc., HT-29 cells, etc., CD133+	Transferrin/transferrin receptor	wtp53 gene	Liposomal delivery complex	Kim et al., 2014b
6.	Glioblastoma, N08-74 cells, etc., CD133+	Cetuximab/epidermal growth factor (EGFR)	Cetuximab	Multifunctional magnetic iron-oxide nanoparticles (IONPs)	Kaluzova et al., 2015

Another important study using Cetuximab (binds to an extracellular region of EGFR/EGFRvIII) in GBM CSCs and non-CSCs has provided insight. The Cetuximab-conjugated iron-oxide nanoparticles were used to define their efficacy against tumors in intracranial rodent GBM model. These studies could help in the animal survival after treatment (Kaluzova et al., 2015).

## Future directions

Altogether, the various types of nanomedicine discovered so far by exploring different tumor models are greatly promising. Many of these nanomedicine approaches have high positive impact on the specificity and efficacy of the conventional anti-tumor/anti-CSCs agents. This influence can boost the probabilities of their use in clinics also. However, the development of most effective clinical regimen needs more detailed insights to solve various relevant issues as discussed above. Many nanomedicines have already received clinical approval and seek an urgent attention toward more comprehensive research for their further advancevements. In this virtue, few potential directions are discussed here to get some more attention for the development of most advanced technology.

### Optimal nanomedicine with higher efficiency is essential for regulation of CSCs/tumor growth

Since payload carrying capacity and other relevant issues are crucial in determining the efficacy of any nanomedicine, the development of efficient nanocarriers would improve the therapeutic effectiveness of them. In addition to the identification of CSCs drug resistance mechanistic and focusing on identification of more CSC specific targeting molecules/markers, designing/synthesis and optimization of nanocarriers application should be of more importance in this context (Table 2: A summary of innovative and potential drug delivery systems for efficient CSCs elimination). Various issues seek immediate attention to developing more efficient nanomedicines which are discussed as followings:

### Specificity or targeted nanocarriers related issues

Deposition of anti-tumor/CSCs agents should be precisely in desired tumor sites/CSCs subpopulation, and there are increasing reports on various types of targeted nanoparticles based anti-tumor therapeutic approaches, which have been encouraging researcher to develop more efficient similar nanoparticle based delivery systems (Wang K. et al., 2012). But these methods suffer from many problems such as modification of nanoparticles (to make them targeted)would add further complexities to the synthesis processes, increase production cost and some regulatory barriers may need to be overcome (Cheng et al., 2012). Further, few research groups have criticized the ability of targeted nanomedicine to deliver the anti-tumor/CSCs moieties into desired sites. It is argued that addition of targeting moieties would compromise the stealth feature of nanoparticles and may suffer from enhanced clearance rate by host clearing system (McNeeley et al., 2007). For example, non-targeted liposomal nanoparticles may exert higher accumulation potential comparable to functionalized-liposomal nanoparticles due their longer circulation time and greater EPR (McNeeley et al., 2007). Another paradox is associated with the high avidity of nanoparticle which is believed to be advantageous but targeted high avidity nanoparticles have been demonstrated to exert reduced penetration in the deep tumor layers (Lee et al., 2010). It is believed that targeted nanoparticles may find it difficult to reach all the CSCs which are residing in the necrotic areas of tumors (Keith and Simon, 2007) and targeted nanoparticles would be more useful against those cancers where they can easily reach to the CSCs, e.g., hematological malignancies. Other difficulties appear due to the non-expression of most common cancer cell surface markers (e.g., HER2 receptor, Transferrin receptor) by CSCs and common expression of most cell surface markers by both the CSCs and healthy stem cells. The common expression profile makes the use of present targeted-nanocarriers less specific which can elicit undesired side-effects (Xia, 2014). These pitfalls indicate the end of the most elaborated definition of CSCs specificity and identification of more stringent anti-CSCs specific marker which would help us in developing most effective targeted nanomedicines.

#### Enhanced cellular intake would be required to increase the potency of nanocarriers

As discussed above rationally designed nanocarriers holds the key for efficient delivery of anti-CSCs agents to the specific sites with longer retention/circulation time and sufficient cellular internalization that can completely eradicate tumors. Rationale designing requires the development of intelligent and versatile delivery systems (e.g., nanoparticle with the capability to respond to the tumor microenvironmental stimuli). One good example could be given as PEGylation (and other hydrophilic modifications) which are proposed to enhance the stability, reducing non-specific protein interactions and also retard the clearance through immune cells (Knop et al., 2010). Unfortunately, PEGylation has been shown to impede the cellular uptake of nanoparticles and cause limited intracellular trafficking that limits their anti-CSCs activities (Mishra et al., 2004). However, efforts are made to overcome these limitations such as PEG moieties should be detached from the nanoparticles after cellular intake by using specific microenvironmental stimuli within tumor cells. For example, there are nanoscale changes in neoplastic disorders such as pH changes and altered expression of matrix metalloproteinases (Hanahan and Weinberg, 2011; Huang et al., 2013; Mura et al., 2013). For example, phospholipids attached to cell penetrating peptides and further coated with pH sensitive PEG. This PEG coating degrades at low pH and liposomes are taken up by cells through their cell-penetrating peptide moieties (Kale and Torchilin, 2010). Other than pH change, proteases and MMP-2 also have been reported to be of use in developing similar environment stimuli-sensitive nanoparticles (Kessenbrock et al., 2010). The micellar nanoparticles are responsive to MMP-2 can form a micelle-plex with siRNA by using a copolymer of PEG/PCL. The PEG/PCL attached through MMP-2 sensitive peptide bridge shown enhanced intracellular intake of micellar-plex due to exposure of cationic peptide polyarginine (r9) after removal of PEG shell in the tumor microenvironment (Wang H. X. et al., 2014). To be noticed, the tumor microenvironment sensitive nanocarriers were equally efficient in getting accumulated (passive) within tumor without having any significant EPR effect on their clearance as compared to non-degradable PEGylated nanoparticles. Similar concept for microenvironment responsive nanocarriers and their interactions with CSCs are not vastly evaluated and need to be explored in more elaborated manner.

### Nanocarriers with the capabilities of penetrating into the deepest interior population are essential for enhancement of their anti-CSCs activities

There are two anatomically distinct regions in tumor microenvironment or niche termed as *outer perivascular region* and *interior hypoxic regions* which are mostly populated by CSCs (Li Z. et al., 2009; Charles et al., 2010; Mohyeldin et al., 2010). For example, aldehyde dehydrogenase (ALD) labeled highly proliferating epithelial-like breast CSCs are demonstrated to be located in the interiors of tumors (Liu et al., 2014).

The accessibility to the peripherally located CSCs by therapeutic agents due to the fully developed vasculature in these regions make them an easy target. Whereas, another interior regions remain poorly vascularised and characterized by hypoxic environment due to the immature vasculature and immense interstitial matrix causing the reduced penetration of therapeutic agents and comparable higher survival rate of CSCs in them (Mohyeldin et al., 2010). The rationale to improve penetration capability of nanocarriers loaded with anti-CSCs agents could be a better option to target these CSCs. In fact, various methods are demonstrated to improve the penetration and subsequent retention of nanocarriers based drugs in the desired tumor tissues. For example, PEGylation, manipulating surface charge of nanoparticles, particle size, and tissue penetrating peptide attachments are some good examples (Kim et al., 2010; Cabral et al., 2011; Jokerst et al., 2011; Ruoslahti, 2012). Another smart delivery system may involve changeable properties (e.g., structure, size) according to variables in the microenvironment such as low pH, low oxygen concentration, and high concentration of proteases in the interiors of the tumor microenvironment. These smart nanoparticles would be carrying multiple components and can control the release and penetration/accumulation of therapeutic agents in tumor niche. Development of pH sensitive liposome-based dendrimer nanocarriers have been a good example of this approach showing enhanced circulation and accumulation in the tissue. After initial accumulation in tumor tissues, these nano-assemblies were capable of penetrating tightly packed tumor cellular microenvironment (containing a dense array of extracellular matrix) resulting in increased drug intake by tumor cells even in the distant regions (Sun Q. et al., 2014).

### Genome editing aided nanomedicine for CSCs eradication

In addition to existing RNAi approaches (e.g., siRNA and miRNA therapeutic agents) more efficient gene editing strategies might open a new door of hope for generating most effective anti-CSCs remedies. The RNAi-based approaches rely upon the RNA degradation/inhibition of translation of genes supporting CSCs survival/function without having any effect on the gene expression as such (Castanotto and Rossi, 2009). Therefore, disease seeking permanent shut down of specific gene expression may not be benefitted from this approach. Also, RNAi-related poor specificity and other off-target effects may also decrease the overall therapeutic value of these strategies (Mittal, 2004; Jackson and Linsley, 2010). Genome editing technology provides a platform for the development of newer and better approach. It involves programmable nucleases [e.g., meganucleases, zinc-finger nucleases (ZFNs), transcription activation like effector nucleases (TALENs), and the clustered regularly interspaced short palindromic repeat (CRISPR) associated nuclease Cas9] (Wolfe et al., 2000; Bibikova et al., 2003; Smith et al., 2006; Christian et al., 2010; Miller et al., 2011; Cong et al., 2013; Ran et al., 2013; Boissel et al., 2014) for editing genome in diseased cells/tissues to cause inactivation/correction of malfunctioning gene(s) due to mutations, generation of proactive mutations and addition of therapeutically effective transgenes (Boettcher and McManus, 2015; Cox et al., 2015). Most importantly, CRYPT based strategies are grabbing more attentions of the researchers across the globe for the study of gene functions, genomic rearrangement, disease progression in both cancer and other disorders, and corrections of inhered genetic disorders (Ran et al., 2013; Cox et al., 2015). CRISPR/Cas9 based approached are more efficient in disruption of gene function in targeted gene knockdown as compared to RNAi-based approaches which rely upon protein depletion related cellular inadequacies (Qi et al., 2013; Shalem et al., 2014). Use of CRISPR-based genome editing technology for the suppression of ABC transporters cassette through targeted nanocarriers would be an efficient method for improved accumulation of anti-CSCs drug molecules (Platt et al., 2014; Aida et al., 2015). For instance, BMP-4 gene addition by genome editing technology promoted differentiation ofCD133+ HCC CSCs while blocking it self-renewal and make them sensitive to the chemotherapeutic agents (Zhang L. et al., 2012).

Although promising the delivery of various editing systems to the particular cell or sites would remain major barriers in the way of their clinical translation.

Due to their low packaging capacity, high expression of nucleases and marked immunogenic effect, the virus-based delivery system are significantly avoidable in the most clinical setting. On the contrary, polymer or liposomal-based methods seems to grab more attention in clinical settings due to their comparatively small toxicity range (Bessis et al., 2004; Wu et al., 2010; Cox et al., 2015; Zuris et al., 2015).

### Improvements in the immunotherapy of CSCs

Different types of immune cells (e.g., T cells, macrophages, natural killer cells, etc.) are known to affect the CSCs (both inhibitory/stimulatory effects) in tumor microenvironment along with other types of cells such as mesenchymal stem cells, tissue associated fibroblast, and endothelial cells (Korkaya et al., 2011). Recent advancements in the identification of tumor cells and infiltrating immune cells have raised the potential use of immune therapeutics in clinics (Pan et al., 2015).

Endogenous immune response regulators (e.g., cell surface molecules) functions as immunity checkpoint and can regulate autoimmune responses through their regulatory effects on the various co-inhibitory signaling pathways (Nirschl and Drake, 2013). However, in the case of cancer, these inhibitory pathways facilitate tumor immune resistance (Naidoo et al., 2014). Various research groups have demonstrated major immunoinhibitory pathways. For instance, programmed cell death-1 (PD-1)/PD-L1 axis, and the cytotoxic T-lymphocyte antigen 4 (CTLA-4)/B7 axis, which contribute cancer cell protection through their suppressive role in tumor microenvironment and negatively regulate cancer cell eradication by immune destruction methods (Pardoll, 2012; Lyford-Pike et al., 2013). Since stem cells are immune-privileged and have an active role in immuno-regulations In CSC niche. The secretion of various paracrine factors by CSCs can reciprocally modulate the immune cells (Frank and Sayegh, 2004; Le Blanc et al., 2004; Le Blanc and Ringden, 2007; Schatton et al., 2008; Maccalli et al., 2014). IT is demonstrated that CSC can negatively regulate T-cell activities (Schatton and Frank, 2008; Schatton et al., 2010). By expression of the chemoresistance determining factor ABCB5 a novel type of CSCs has been identified that is known as malignant melanoma initiating cells (MMIC). The MMICs preferentially express PD-1 and B7.2 but significant decrease in the expression level of PD-L1 compared to ABCB5-cells (Schatton and Frank, 2008). Reports are showing the clinical use of anti-PD-1/PDL-1 monoclonal antibodies in various cancers including melanomas and lung cancer (Sharma et al., 2011; Topalian et al., 2012), and in refractory Hodgkin's disease (Ansell et al., 2014). In these clinical studies, few patients had considerably prolonged responses in comparison to cytotoxic/targeted therapies, and as per assumption activation of T-cell may decline by PD-1/PD-L1 expression in tumor cells. For instance, head and neck carcinoma cells are reported to have high expression level of PD-1/PD-L1 (Lee and Sunwoo, 2014). Thus, it is postulated that in future clinical trials assessment of CSCs' ability to respond immune blockade checkpoint can be an important determining factor. Also, the combined use of immune-checkpoint therapies and CSCs targeting immunotherapy (e.g., vaccine) may be a useful tool to enhance their clinical utility.

Studies reported that EMT program has positive effect o the expression level of CD90 and EphA4 which are known to participate in interactions among CSCs and tumor-associated monocytes and macrophages (TAMs) and these TAMs create a CSCs niche causing enhanced CSCs activities of carcinoma cells (Lu et al., 2014). In another study, breast cancer cells were shown to have resistance against autologous/allogeneic natural killer cells due to reduced expression of MICA and MICAB (two ligands for the stimulatory receptor NKG2D) (Wang B. et al., 2014). In a mouse study, the potential of ALDH1A1-based immunotherapy (via adoptive cell therapy causing the elimination of ALDH bright + population) have been demonstrated to be of therapeutic importance (Visus et al., 2011).

Implementation of CSCs-based dendritic cell vaccine has also been useful in developing anti-CSCs immunity. For example, DV vaccination by neurosphere showed stronger anti-tumor effects on in comparison to conventionally grown cells in a mouse glioma model (Ning et al., 2012; Toda, 2013). In similar studies to evaluate the preventive effect of CSC-based vaccination on the liver metastasis development in a rat colon cancer model shown a significant reduction in the tumor volume incidence (Duarte et al., 2013).

Nanoparticles are useful as a carrier for vaccine antigen and have been promising in the development of cancer immunotherapy (Park et al., 2013; Goldberg, 2015). The rationally designed nanoparticles have been shown to reduce the accumulation of TAMs or can destroy TAMs which is defined as an essential component of CSCs niche (Leuschner et al., 2011; Zhu et al., 2013). Another report indicates more encouraging results showing a role as an adjuvant for the nanoparticles which can stabilize the vaccine antigens boost the response of antigen-specific CD8+ T cells and therefore enhancing the anti-cancer immunity. Further, changing the size, charge and hydrophobic characteristics of nanoparticles and equip them with suitable targeting moieties may improve their accessibility to the antigen presenting cells (APCs) and modulate the immune response to an antigen resulting to improved anti-cancer therapy (Cruz et al., 2012). Also, use of high-through screening methods may help in identifying new and more accurate anti-CSCs antigens to improve the development of more targeted nanomedicines. A more interesting combination would be the nanotechnology and immunotherapeutic holding greater promises to achieve success in most efficient anti-cancer remedies.

## Conclusion

The article entails various approaches to tackle CSCs/tumor cells while describing in-depth knowledge of their biological parameters and significance in tumors. The primary focus was on the use of different nanotechnology-based therapeutic approaches for the effective eradication of CSCs within the tumor to completely cure cancers. Nanomedicines may be the treatment of choice for all the different types of cancer due to their excellent efficacy in penetration, specific retention and killing of tumor cells/CSCs. However, a lot of many issues which should be dealt in priority to ensure the maximum benefit from the recent advancements in the field of nanomedicine is essential. Also, use of multidisciplinary tactics for the enhancement of the efficiency of both conventional and nanotechnology based and cancer therapeutics regime would be substantial asset. In short, nanomedicine is the future of cancer treatment and would require more in-depth knowledge of basic information of cancer cells and other allied subjects (such as chemical synthesis of optimal nanocarriers) to improve the existing barriers in this field further.

## Author contributions

VS, manuscript conceptualization, design, writing, editing, and final approval; AS, editing, formatting, artwork designing; RC, Conceptualization, editing, and final discussion.

### Conflict of interest statement

The authors declare that the research was conducted in the absence of any commercial or financial relationships that could be construed as a potential conflict of interest.
